# P06 Management challenges and treatment options of difficult-to-treat organisms in patients with complex medical devices: a case of vancomycin-resistant *Enterococcus* infection in a patient with a left ventricular assist device

**DOI:** 10.1093/jacamr/dlae136.010

**Published:** 2024-08-23

**Authors:** Joshua Hrycaiczuk, Bazga Ali

**Affiliations:** Public Health Wales, UK; Public Health Wales, UK

## Abstract

**Background:**

Medical devices are becoming increasingly sophisticated. Inserting prosthetic devices into a patient has an inherent risk of infection and as the complexity of devices increases the management of infective complications becomes more problematic. Over the last 40 years the incidence of cardiac device-related infective endocarditis has risen more than fourfold.^1^ This risk is compounded by the increasing prevalence of antimicrobial resistance limiting treatment options.

**Clinical case:**

A 65-year-old man had a left ventricular assist device (LVAD) inserted for severe ischaemic cardiomyopathy. An LVAD is an electromechanical pump placed into the left ventricle; it supports it in maintaining cardiac output acting as either a bridge to recovery or transplant, or to extend the patient’s quality and duration of life.^2^ This insertion was complicated by vancomycin-susceptible *Enterococcus faecium* bacteraemia with PET scan demonstrating abnormal update within the LVAD system. He was treated with 6 weeks of IV vancomycin and was discharged to the outpatient setting to continue 6 weekly dalbavancin for the next 12 months. Nine months into treatment, bacteraemia recurred and susceptibilities demonstrated the organism had become resistant to vancomycin. Long-term, non-toxic treatment options were needed as, due to the complex nature of the LVAD device, removal was not viable. Treatment choices were limited, and because of the toxic effects associated with long-term linezolid use, daptomycin was chosen as a daily OPAT option. Subsequent daptomycin MIC testing suggested this would be ineffective (MIC=12) and his treatment was switched to minocycline plus rifampicin. Further testing demonstrated rifampicin resistance (MIC=32) and minocycline partial resistance (MIC=4). The patient remains stable and asymptomatic in the community on minocycline whilst further long-term therapeutic options are considered to suppress infection in this challenging case.

**Conclusions:**

This case highlights the growing problem of managing chronic infections with resistant organisms in patients with irremovable devices. Treatment options specifically for vancomycin-resistant *Enterococcus* are limited, especially when considering a case where the patient requires prolonged outpatient antibiotics to sustain meaningful quality of life. Although new antimicrobials (contezolid and delpazolid) are in development for VRE, these do not have a novel mechanism of action.^3^ This case demonstrates the importance of developing novel therapeutic options to combat resistant organisms and the significance of minimizing the development and spread of antimicrobial resistance.
